# Real-Time Single Image Depth Perception in the Wild with Handheld Devices

**DOI:** 10.3390/s21010015

**Published:** 2020-12-22

**Authors:** Filippo Aleotti, Giulio Zaccaroni, Luca Bartolomei, Matteo Poggi, Fabio Tosi, Stefano Mattoccia

**Affiliations:** Department of Computer Science and Engineering, University of Bologna, 40136 Bologna, Italy; filippo.aleotti2@unibo.it (F.A.); giulio.zaccaroni@studio.unibo.it (G.Z.); luca.bartolomei4@studio.unibo.it (L.B.); fabio.tosi5@unibo.it (F.T.); stefano.mattoccia@unibo.it (S.M.)

**Keywords:** monocular depth estimation, deep learning, mobile systems, smartphone

## Abstract

Depth perception is paramount for tackling real-world problems, ranging from autonomous driving to consumer applications. For the latter, depth estimation from a single image would represent the most versatile solution since a standard camera is available on almost any handheld device. Nonetheless, two main issues limit the practical deployment of monocular depth estimation methods on such devices: (i) the low reliability when deployed in the wild and (ii) the resources needed to achieve real-time performance, often not compatible with low-power embedded systems. Therefore, in this paper, we deeply investigate all these issues, showing how they are both addressable by adopting appropriate network design and training strategies. Moreover, we also outline how to map the resulting networks on handheld devices to achieve real-time performance. Our thorough evaluation highlights the ability of such fast networks to generalize well to new environments, a crucial feature required to tackle the extremely varied contexts faced in real applications. Indeed, to further support this evidence, we report experimental results concerning real-time, depth-aware augmented reality and image blurring with smartphones in the wild.

## 1. Introduction

Depth perception is an essential step to tackling real-world problems, such as robotics [1], autonomous driving [2], many other tasks involving 3D reconstruction [3] and cultural heritage applications [4]. Consequently, some well-known sensors exist for this purpose. Among them, active sensing techniques such as time-of-flight (ToF), structured light and LiDAR are often deployed in the application domains mentioned before. However, they struggle when dealing with some typical consumer applications. On the one hand, ToF and structured light are mostly suited for indoor environments and allow, for instance, for human pose estimation [5]. On the other hand, conventional LiDAR technology, frequently used for autonomous driving and other tasks is too cumbersome and expensive for deployment with relatively cheap and lightweight consumer handheld devices. Nonetheless, it is worth noting that active sensing technologies, mostly suited for indoor environments, are sometimes integrated into high-end devices. For instance, they are present in the 2020 Apple iPad Pro and iPhone 12 Pro.

Therefore, camera-based technologies are often the only viable strategies to infer depth in consumer applications, and among them, well-known methodologies are structured-light and stereo vision. The former typically requires an infrared camera and a specific pattern projector, making it unsuitable for environments flooded by sunlight. The latter requires two appropriately spaced and synchronized cameras. Some recent high-end smartphones or tablets feature one or both technologies, although they are not yet widespread enough to be considered standard equipment. Moreover, the distance between the cameras (baseline) is necessarily narrow for stereo systems, limiting the depth range to a few meters away.

On the other hand, with the spread of deep-learning, recent years witnessed the rising of a different strategy to infer depth using a single standard camera available substantially in any consumer device. Compared to previous technologies for depth estimation, such an approach would enable one to tackle all the limitations mentioned before. Nonetheless, single-image depth estimation is seldom deployed in consumer applications due to two main reasons. The first one concerns the low reliability of state-of-the-art methods when tackling depth estimation in the wild dealing with unpredictable environments not seen at training time, as it occurs when targeting a massive number of users facing heterogeneous environments. The second reason concerns the constrained computational resources available in handheld devices, such as smartphones and tablets, deployed for consumer applications. In fact, despite the steady progress in this field, the gap with systems leveraging high-end GPUs is (and always will be) significant, since power requirements heavily constrain handheld devices. Despite the limited computational resources available, most consumer applications call for real-time performance.

**Figure 1:**
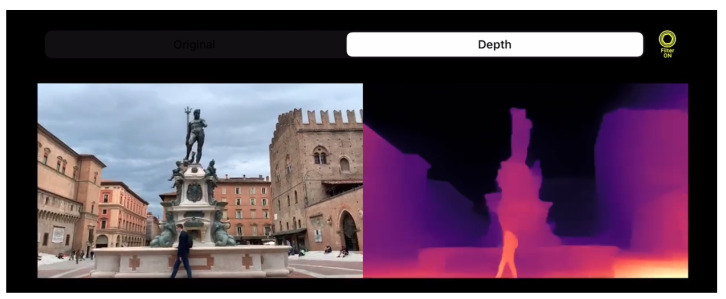
Depth perception in the wild with a mobile application. Single image depth perception in the wild at nearly 60 FPS with an iPhone XS and the PyDNet [6] network.

Arguing these facts, in this paper, we deeply investigate both issues outlined so far. In particular, we show how to tackle them by leveraging appropriate network designs and training strategies, and outline how to map them on off-the-shelf consumer devices to achieve real-time performance, as shown in Figure 1. Specifically, we propose a strategy to distill knowledge using standard still images from a robust yet complex monocular network unsuited for real-time performance and apply this methodology to lightweight and fast networks to achieve the desired performance on mobile devices. Indeed, our extensive evaluation highlights the resulting network’s ability to robustly generalize to unseen environments, a crucial feature to tackle the heterogeneous contexts faced in real consumer applications.

The paper is organized as follows. At first, we review previous works about monocular depth estimation on high performance and mobile devices; then we describe the framework that allows us to train a model, even a lightweight one, to be robust when deployed in the wild. Eventually, we evaluate a subset of monocular networks proposed in the literature on three benchmarks, deploying such models on mobile smartphones. Finally, we report how achieving real-time single image depth estimation at the edge can be effectively exploited to tackle two well-known applications: depth aware augmented reality and blurring.

## 2. Related Work

Monocular depth estimation. Even if depth estimation from multiple views has a long history in computer vision, depth from a single image [7,8] started being considered feasible only with the advent of deep learning techniques. Indeed, obtaining depth values starting from a single image is an ill-posed problem, since an infinite number of real-world layouts may have generated the input image. However, learning-based methods, and in particular deep learning, proved to be adequate to face the problem. Initially were proposed supervised approaches [9,10]. On this track, He et al. [11] faced the ambiguity caused by varying focal lengths across different images. However, the need for ground truth measurements represents a severe restraint since an active sensor, as a LiDAR, together with specific manual post-processing is required to obtain such data. Therefore, effective deployment of supervised approaches is burdensome both in terms of time and costs. To overcome this limitation, solutions able to learn depth supervised only by images are incredibly appealing, and nowadays, several methods rely on simple stereo pairs or monocular video sequences for training. Among these, ref. [12] is the first notable attempt leveraging stereo pairs, eventually improved exploiting traditional stereo algorithm [13,14], visual odometry supervision [15,16] or 3D movies [17]. On the other hand, methods leveraging monocular videos do not even require a stereo camera at training time, at the cost of learning depth estimation up to a scale factor. Consider Zhou et al. [18] proposing to learn both depth and camera ego-motion; more recent methods propose to apply direct visual odometry [19] or ICP [20] strategies to improve predictions. Additional cues have been used by more recent works, such as optical flow [21,22,23,24] and semantic segmentation [25]. Finally, it is worth noting that the two supervisions coming from stereo images and monocular sequences can be combined [26]. Some works also focus on understanding which the cues that neural networks exploit to infer depth from single images are [27].

Depth estimation on mobile systems. Mobile devices are ubiquitous, and deep learning opened many application scenarios [28]. Although sometimes server-side inference is unavoidable, maintaining the computation at the edge is highly beneficial. It allows one to get-rid of privacy issues and the need for tailored datacentersm allowing one to reduce costs and improve scalability. Moreover, although not critical for most consumer scenarios, full on-board processing does not dictate an Internet connection. Despite limited computing capabilities, mostly constrained by power consumption issues and typically not comparable to those available in standard laptops or PCs, some authors proposed deep networks for depth estimation suited for mobile devices too. These works targeted stereo [29] and monocular [6,30] setups. Moreover, some authors proposed depth estimation architectures tailored for specific hardware setups, such as those based on dual-pixel sensors available in some recent Google’s smartphones, as reported in [31,32].

## 3. Problems and Requirements

The availability of more and more powerful devices paves the way for complex and immersive applications, in which users can interact with the nearby environment. As a notable example, augmented reality can be used to display interactive tools or concepts, avoiding the need to build a real prototype and thus cutting costs. For this and many other applications, obtaining accurate depth information with a high frame rate is paramount to further enhancing the interaction with the surrounding environment, even with devices devoid of active sensors. Almost any modern handheld device features at least a single camera and an integrated CPU within—typically, an ARM-based system-on-chip to cope with the constrained energy budget of such devices. Sometimes, especially in most new ones, a neural processing unit (NPU) devoted to accelerating deep neural networks is also available. Inevitably, the resulting overall computational performance is far from conventional PC-based setups, and the availability of an NPU only partially fills this gap. Given these constraints, single image depth perception is rather appealing, since it could seamlessly deal with dynamic contexts, whereas other techniques such as structure from motion (SfM) would struggle. However, these techniques are computationally demanding, and most state-of-the-art approaches would not work with the computational resources available in handheld devices. Moreover, regardless of the computing requirements, training the networks for predictable target environments is not feasible for consumer applications. Thus the depth estimation network shall be robust to any faced deployment scenarios and possibly invariant to the training data distribution. A client–server approach would soften some of the computational issues, although with notable disadvantages—the need for an Internet connection and a poorly scaling of the whole overall system when the number of users increases.

To get rid of all the issues mentioned above and to deal with practical applications, we will describe next how to achieve real-time and robust single image depth perception on low-power architectures found in off-the-shelf handheld devices.

## 4. Framework Overview

In this section, we introduce our framework aimed at enabling single image depth estimation in the wild with mobile devices, devoting specific attention to iOS and Android systems. Before the actual deployment on the target handheld device, our strategy requires an offline training procedure typically carried out on power unconstrained devices. We will discuss in the reminder the training methodology, leveraging knowledge distillation, deployed to achieve our goal in a limited amount of time and the dataset adopted for this purpose. Another critical component of our framework is a lightweight network enabling real-time processing on the target handheld devices. Purposely, we will introduce and thoroughly assess the performance of state-of-the-art networks fitting this constraint.

### 4.1. Offline Training

As for most learning-based monocular depth estimation models, our proposal is trained offline on standard workstations, equipped with one or more GPUs, or through cloud processing services. In principle, depending on the training data available, one can leverage different training strategies: supervised, semi-supervised and self-supervised training paradigms. Moreover, as done in this paper, cheaper and better-scaling supervision can be conveniently obtained from another network, leveraging knowledge distillation to avoid the need for expensive ground truth labels, through a teacher–student network.

When a large enough dataset providing ground truth labels inferred by an active sensor is available, such as [33,34], (semi-)supervised training is certainly valuable, since it enables one, among other things, to disambiguate difficult regions (e.g., texture-less regions such as walls). Unfortunately, large datasets with depth labels are not available or extremely costly and cumbersome to obtain. Therefore, when this condition is not met, self-supervised paradigms enable one to train with (potentially) countless examples, at the cost of a more challenging training setup and typically less accurate results. Note that, depending on the dataset, a strong depth prior can be distilled, even if there are not available depth labels provided by an active sensor. For instance, refs. [13,14] exploited depth values from a stereo algorithm, and [35] relied on a SfM pipeline. Finally, supervision can be distilled from other networks as well, for the stereo [36] and monocular [37] setups. The latter is the strategy followed in this paper. Specifically, we use as the teacher the MiDaS network proposed in [17]. This strategy allows us to speed-up the training procedure of the considered lightweight networks significantly, since doing this from scratch according to the methodology proposed in [17] would take a far longer amount of time (weeks vs. days), being mostly bounded by the demanding generation of proxy labels. Moreover, it is worth noting that given a reliable teacher network, pre-trained in a semi or self-supervised manner, such as [17], it is straightforward to distill an appropriate training dataset, since any collection of images is potentially suited to this aim. We will describe next the training dataset used for our experiments made of a bunch of single images belonging to well-known popular datasets.

### 4.2. On-Device Deployment and Inference

Once outlined the training paradigm, the next issue concerns the choice of a network capable of learning from the teacher how to infer meaningful depth maps, and at the same time, be able to run them in real-time on the target handheld devices. Unfortunately, only a few networks described next potentially fulfill these requirements, in particular, considering the ability to run in real-time on embedded systems.

Having identified and trained a suitable network, the mapping on a mobile device is nowadays quite easy. In fact, there exist various tools that, starting from a deep learning framework such as PyTorch [38] or TensorFlow [39], can export, optimize (e.g., perform weight quantization) and execute models, even leveraging mobile GPUs [40] on principal operating systems (OS). In some cases, the target OS exposes utilities and tools to improve the performances further. For instance, starting from iOS 13, neural networks deployed on iPhones can use the GPU or even the Apple Neural Engine (ANE) thanks to Metal and Metal Performance Shaders (MPS), thereby largely improving the runtime performances. We will discuss in the next section how to map the networks on iOS and Android devices using high-level development frameworks.

## 5. Lightweight Networks for Single Image Depth Estimation

In the remainder, when not strictly required, we refer to depth and inverse depth interchangeably. According to the previous discussion, only a subset of the state-of-the-art single image depth estimation networks fits our purposes. Specifically, we consider the following publicly available lightweight architectures: PyDNet [6], DSNet [25] and FastDepth [30]. Moreover, we also include a representative example of a large state-of-the-art network MonoDepth2, proposed in [26]. It is worth noticing that other and more complex state-of-the-art networks, such as [13], could be deployed instead within the proposed framework. However, this might come at the cost of higher execution time on the embedded device, and potentially, overhead for the developer in case of custom layers not directly supported by the mobile executor (e.g., the correlation layer used in [13]).

**MonoDepth2.** An architecture deploying a ResNet encoder, proposed initially in [41], made of 18 feature extraction layers, shrinking the input by a factor of $\frac{1}{32}$. Then, the dense layers are replaced in favour of a decoder module, able to restore the original input resolution and output an estimated depth map. At each level in the decoder, 3×3 convolutions with skip connections are performed, followed by a 3×3 convolution layer in charge of depth estimation. The resulting network can predict depth at different scales, counting overall 14.84 M parameters. It is worth to notice that in our evaluation we do not rely on ImageNet [42] pre-training for the encoder for fairness to other architectures not pre-trained at all.

**PyDNet.** This network, proposed in [6], features a pyramidal encoder-decoder design able to infer depth maps from a single RGB image. Thanks to its small size and design choices, PyDNet can run on almost any device including low-power embedded platforms [43], such as the Raspberry Pi 3. In particular, the network exploits 6 layers to reduce the input resolution at $\frac{1}{64}$, restored in the depth domain by 5 layers in the decoder. Each layer in the decoder applies 3×3 convolutions with 96,64,32,8 feature channels, followed by a 3×3 convolution in charge of depth estimation. Notice that, to keep low the resources and inference time, top prediction of PyDNet is at half resolution, so the final depth map is obtained through an upsampling operation. We adopt the mobile implementation provided by the authors, publicly available online (https://github.com/FilippoAleotti/mobilePydnet), which differs from the paper network by small changes (e.g., transposed convolutions have been replaced by upsampling and convolution blocks). The overall network counts 1.97 M parameters.

**FastDepth.** Proposed by Wofk et al. [30], this network can infer depth predictions at 178 fps with an NVIDIA Jetson TX2 GPU. This notable speed is the result of design choices and optimization steps. Specifically, the encoder is a MobileNet [28], thus suited for execution on embedded devices. The decoder consists of 6 layers, each one with a depth-wise separable convolution, with skip connections starting from the encoder (in this case, features are combined with addition). However, it is worth observing that the highest frame rate previously reported is achievable only exploiting both pruning [44] and hardware-specific optimization techniques. In this paper, we do not rely on such strategies for fairness with other networks. The whole network counts 3.93 M parameters.

**DSNet**. This architecture is part of ΩNet [25], an ensemble of networks predicting not only the depth of the scene starting from a single view but also the semantic segmentation, camera intrinsic parameters and if two frames are provided, the optical flow. In our evaluation we consider only the depth estimation network DSNet, inspired by PyDNet, which contains a feature extractor able to decrease the resolution by $\frac{1}{32}$, followed by 5 decoding layers to infer depth predictions starting from the current features and previous depth estimate. In the original architecture, the last decoder also predicts per-pixel semantic labels through a dedicated layer, removed in this work. With this change, the overall network counts 1.91 M of parameters, 0.2 M fewer than the original model.

## 6. Datasets

In our evaluation, we use four datasets. At first, we rely on the KITTI dataset to assess the performance of the four networks when trained with the standard self-supervised paradigm deployed typically in this field [26]. Then, we re-train from scratch the four networks using the paradigm previously outlined, distilling proxy labels by employing the pre-trained MiDaS network [17] made available by the same authors. For this task, we use a novel dataset, referred to as WILD, described next. We then evaluate the networks trained according to this methodology on the TUM RGBD [45] and NYUv2 [46] dataset to assess their generalization capability.

KITTI. The KITTI dataset [47] contains 61 scenes collected by a moving car equipped with a LiDAR sensor and a stereo rig. Following [26], we select a split of 697 images for testing, while 39,810 and 4424 images are used respectively for preliminary training and validation purpose. Moreover, we use it to assess the generalization capability of the networks in the wild during the second part of our evaluation.

WILD. The Wild dataset (W), introduced in this paper, consists of a mixture of Microsoft COCO [48] and OpenImages [49] datasets. Both datasets contain a large number of Internet photos, and they do not provide depth labels. Moreover, since neither video sequences nor stereo pairs are available, they are not suited for conventional self-supervised guidance methods (e.g., SfM or stereo algorithms). On the other hand, they cover a broad spectrum of various real-world situations, allowing to face both indoor and outdoor environments, deal with everyday objects and various depth ranges. We select almost 447,000 frames for training purposes. Details concerning the WILD dataset are available at this link: https://github.com/FilippoAleotti/mobilePydnet.

Then, we distilled the supervision required by our networks with the robust monocular architecture proposed in [17] with the weights publicly available. Such a network provides as output an inverse depth up to a scale factor. We point out once again that our supervision protocol has been carefully chosen mostly for practical reasons. It takes a few days to distill the WILD dataset by running MiDaS (using the publicly available checkpoints) on a single machine. On the contrary, to obtain the same data used to train the network as in [17], it would require an extremely intensive effort. Doing so, we can scale better: since we trust the teacher, we could, in principle, source knowledge from various and heterogeneous domains on the fly. Of course, the major drawbacks of this approach are evident: we need an already available and reliable teacher, and the accuracy of the student is bounded to the one of the teacher. However, we point out that the training scheme proposed in [17] is general, so it can also be applied in our case, and that we already expect a margin with state-of-the-art networks due to the lightweight size of mobile architectures considered. For these reasons, we believe that our approach is beneficial to source a fast prototype than can be improved later leveraging other techniques if needed. This belief is supported by experimental results presented later in the paper.

TUM RGBD. The TUM RGBD (3D Object Reconstruction category) dataset [45] contains indoor sequences framing people and furnitures. We adopt the same split of 1815 images used in [35] for evaluation purposes only.

NYUv2. The NYUv2 dataset [46] is an indoor RGBD dataset acquired with a Microsoft Kinect. It provides more than 400k raw depth frames and 1449 densely labelled frames. As for the previous dataset, we adopt the official test split containing 649 images for generalization tests.

## 7. Mapping on Mobile Devices

Since we aim at mapping single image depth estimation networks on handheld devices, we briefly outline here the steps required to carry out this task. Different tools are available according to both the deep learning framework and the target OS. For instance, with TensorFlow models, weights are processed using tf-coreml converter in case of iOS deployment or TensorFlow Lite converter when targeting an Android device. This conversion is possible seamlessly if the networks consist of standard layers, but it is not straightforward in cases of custom modules (e.g., correlation layers as in monoResMatch [13]). Although these tools typically enable one to perform weight quantization during the model conversion phase to the target environment, we refrained from applying quantization to maintain the original accuracy of each network. In the experimental results section, we provide execution time mapping the networks on mobile devices following the porting strategy outlined.

## 8. Experimental Results

In this section, we thoroughly assess the performances of the considered networks with standard datasets deployed in this field. Since it is different from other methods, FastDepth [30] was not initially evaluated on KITTI; we carried out a preliminary evaluation of all other networks on said dataset. Then, we trained from scratch the considered networks according to the framework outlined on the Wild dataset, evaluating their generalization ability. Finally, we show how to take advantage of the depth maps inferred by such networks for two applications particularly relevant for mobile devices.

### 8.1. Evaluation on KITTI

At first, we investigate the accuracies of the considered networks on the KITTI dataset. Since the models were developed with different frameworks (PyDNet and DSNet in TensorFlow, the other two in PyTorch) and trained on different datasets (FastDepth on NYU v2 [46], others on the Eigen [9] split KITTI [47]), we implemented all the networks in PyTorch. This strategy allowed us to adopt the same self-supervised protocol proposed in [26] to train all the models. This choice is suited for the KITTI dataset, since it exploits stereo sequences, enabling one to achieve the best accuracy. Given two images *I* and $I^{†}$, with known intrinsic parameters (*K* and $K^{†}$) and relative pose of the cameras (*R*, *T*), the network predicts depth D, allowing one to reconstruct the reference image *I* from $I^{†}$:

(1) $$\hat{I}=\omega \left(I^{†},K^{†},R,T,K,D\right)$$

where ω is a differentiable warping function.

Then, the difference between $\hat{I}$ and *I* can be used to supervise the network, thereby improving D, without any ground truth. The loss function used in [26] is composed of a photometric error term $p_{e}$ (2) and an edge-aware regularization term $L_{s}$.

(2) $$p_{e}\left(I,\hat{I}\right)=\alpha \frac{\left(1-\mathrm{SSIM}\left(I,\hat{I}\right)\right)}{2}+\left(1-\alpha \right){∥I-\hat{I}∥}_{1}$$

(3) $$L_{s}={∥\delta_{x}D_{t}^{*}∥}_{1}e^{-{∥\delta_{x}I_{t}∥}_{1}}+{∥\delta_{y}D_{t}^{*}∥}_{1}e^{-{∥\delta_{y}I_{t}∥}_{1}}$$

where SSIM is the structure similarity index [50], and $D*=D/\bar{D}$ is the mean normalized inverse depth proposed in [19]. We adopted the M configuration of [26] to train all the models. Doing so, given the reference image $I_{t}$, at training time we also need $\left\{I_{t-1},I_{t+1}\right\}$, which are respectively the previous and the next frames in the sequence, to leverage the supervision from monocular sequences as well. Purposely, a pose network was trained to estimate relative poses between the frames in the sequencem as in [26]. Moreover, per-pixel minimum and automask strategies were used to preserve fine details: the former select the best $p_{e}$ among multiple views according to occlusions, while the latter helps to filter out pixels that do not change between frames (e.g., scenes with a non-moving camera or dynamic objects that are moving at the same speed of the camera), thereby breaking the moving camera in a stationary world assumption (more details are provided in the original paper [26]). Finally, intermediate predictions, when available, are upsampled and optimized at input resolution.

Considering that all the models have been trained with different configurations on different datasets, we re-trained all the architectures while exploiting the training framework of [26] for a fair comparison. Specifically, we ran 20 epochs of training for each model, decimating the learning rate after 15, on the Eigen train split of KITTI. We used Adam optimizer [51], with an initial learning rate of $10^{-4}$, and minimized the highest three available scales for all the network except FastDepth, which provided full-resolution (i.e., 640×192) predictions only. Since the training framework expects a normalized inverse depth as the output of the network, we replaced the last activation of each architecture (if present) with a sigmoid.

Table 1 summarizes the experimental results of the models tested on the Eigen split of KITTI. The top four rows report the results, if available, provided in the original papers, while last three the accuracy of models re-trained within the framework described so far. This test allows for evaluating the potential of each architecture in fair conditions, regardless of the specific practices, advanced tricks or pre-training deployed in the original works. Not surprisingly, the larger MonoDepth2 model performed better than the three lightweight models, showing non-negligible margins on each evaluation metric when trained in fair conditions. Among th latter, although their performances were comparable, PyDNet was more effective with respect to FastDepth and DSNet for most metrics, such as RMSE and δ<1.25.

**Figure 2:**
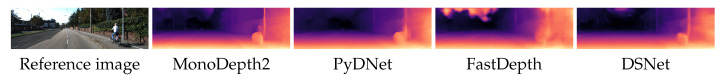
Qualitative results on KITTI. All the models have been trained equally using the framework of [26] on the Eigen split of KITTI.

Figure 2 shows some qualitative results, enabling us to compare depth maps estimated by the four networks considered in our evaluation on a single image from the Eigen test split.

### 8.2. Evaluation in the Wild

**Figure 3:**
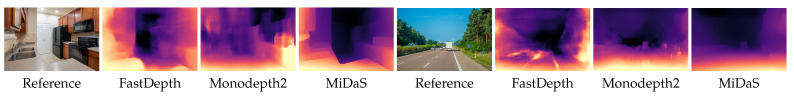
Predictions in the wild. We provide qualitative results for indoor and outdoor Internet images. For each network, we used the checkpoints publicly available. It can be noticed how the networks trained on a single dataset, both in a supervised (FastDepth) and self-supervised (Monodepth2), are not able to generalize well on a different setup. On the contrary, the network trained on various datasets (MiDaS) produced better results. Images from Pexels https://www.pexels.com/.

In the previous section, we assessed the performances of the considered lightweight networks on a data distribution similar to the training one. Unfortunately, this circumstance is seldom found in most practical applications, and typically it is not known in advance where a network will be deployed. Therefore, how can one achieve reliable depth maps in the wild? In Figure 3 we report some qualitative results about original pre-trained networks in different scenarios. Notice that the first two networks have strong constraints about input size (224×224 for [30], 1024×320 for [26]) that these networks internally apply, imposed by how these models have been trained in their original context. Although this limitation, FastDepth (second column) can predict a meaningful result in an indoor environment (first row), not outdoors though (second row). That is not surprising, since the network was trained on NYUv2, which is an indoor dataset. Monodepth2 [26] suffers from the same problem, highlighting that this issue is not concerned with the network size (smaller the first, larger the second) or training approach (supervised the first, self-supervised the second), but it is rather related to the training data. Conversely, MiDaS by Ranftl et al. [17], is effective in both situations. Such robustness comes from a mixture of datasets, collecting about 2M frames covering many different scenarios, used to train a large (∼105 M parameters) and very accurate monocular network.

We leveraged this latter model to distill knowledge and train lightweight models compatible with mobile devices. As mentioned before, this strategy allowed us to use MiDaS knowledge for faster training data generation compared to time-consuming pipelines used to train it, such as COLMAP [52,53]. Moreover, it allowed us to generate additional training samples and thus a much more scalable training set, potentially from any (single) image. Therefore, in order to train our network using the WILD dataset, we first generated proxy labels with MiDaS for each training image of this dataset, clipping the minimum and maximum between 1st and 99th percentiles, although the latter operation is not strictly required. Then, having obtained such proxy labels, we trained the networks using the following loss function:

(4) $$L\left(D_{x}^{s},D_{gt}\right)=\alpha_{l}∥(D_{x}^{s}-D_{gt})∥+\alpha_{s}L_{g}\left(D_{x}^{s},D_{gt}\right)$$

where $L_{g}$ is a gradient loss term minimizing the absolute difference between the predicted depth derivatives (in both directions) and proxy depth derivatives, $D_{x}^{s}$ the prediction of the network at scale *s* (bilinearly upsampled to full resolution) and $D_{gt}$ is the proxy depth. The weight $\alpha_{s}$ depends on the scale *s* and is halved at each lower scale. On the contrary, $\alpha_{l}$ was fixed and set to 1. Intuitively, the $L^{1}$ norm penalizes differences with respect to proxies, while $L_{g}$ helps to preserve sharp edges. We trained the models for 40 epochs, halving the learning rate after 20 and 30, with a batch size of 12 images, with an input size of 640×320. We set the initial value of $\alpha_{s}$ to 0.5 for all networks except for FastDepth, which was set to 0.01. To augment the images, we applied random horizontal flip with 0.5 probability. Additionally, for MonoDepth2 and FastDepth, feature upsampling through the nearest neighbour operator in the decoder phase was replaced with bilinear interpolation. These changes were necessary to mitigate some artifacts found in depth estimations inferred by these networks following the training procedure outlined.

Table 2 collects quantitative results on three datasets: TUM [45] (3D object reconstruction category), KITTI Eigen split [9] and NYU [46]. For each dataset, we first show the results achieved by large and complex networks, MiDaS [17], and the model by Li et al. [35] (using the single frame version), both trained in the wild on a large variety of data. The table also reports results achieved by the four networks considered in our work trained on the WILD dataset, exploiting knowledge distillation from MiDaS. We adopted the same protocol defined in [17] to obtain depths at the same scale of ground truth values from predictions. First and foremost, we highlight how MiDaS performs in general better than [35], emphasizing the reason to distill knowledge from it.

Considering lightweight, compact models, for PyDNet, DSNet and FastDepth we can notice that the margin between them and MiDaS is often non-negligible. A similar behavior occurs for the significantly more complex network MonoDepth2, despite being in general more accurate than other more compact networks, except on KITTI, where it turned out less accurate when trained in the wild. However, considering the massive gap in terms of computational efficiency between compact networks and MiDaS analyzed later, and that MiDaS is not suited at all for real-time inference on the target devices, the outcome reported in Table 2 is not so surprising.

PyDNet was the best model on KITTI when trained in the wild, and also achieved the second-best accuracy on NYU, with minor drops on TUM. Finally, DSNet and FastDepth achieved average performance in general, never resulting in the best on any dataset.

**Figure 4:**
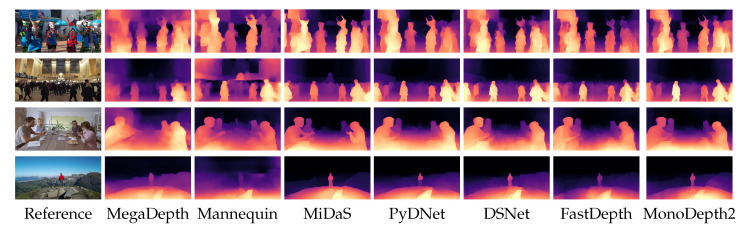
Qualitative results from Internet photos. From left to right, the reference image from Pexels website, and depths from MegaDepth [54], Mannequin [35], MiDaS [17], PyDNet, DSNet, FastDepth and MonoDepth2.

Figure 4 shows some qualitative examples of depth maps processed from Internet pictures by MegaDepth [54], the model by Li et al. [35], MiDaS [17] and the networks trained through knowledge distillation.

**Figure 5:**
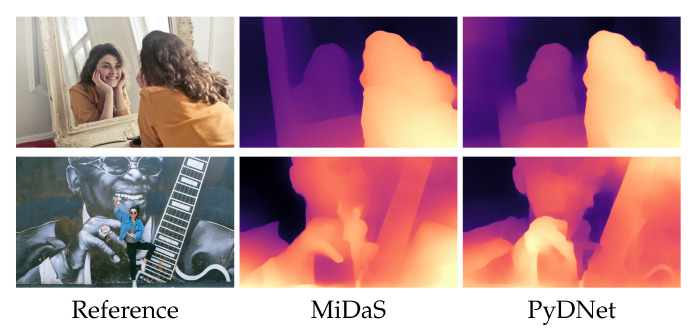
Failure cases. Examples of failure cases of single image depth estimation. From left to right: input image, depth predicted by the teacher and depth predicted by the student.

Finally, in Figure 5, we report some example of failure cases of MiDaS (in the middle column) inherited by student networks. Since both networks failed, the problem is not attributable to their different architectures. Observing the figure, we can notice that such behavior occurs in very ambiguous scenes, such as when dealing with mirrors or flat surfaces with content aimed at inducing optical illusions in the observers.

### 8.3. Performance Analysis on Mobile Devices

Once training the considered architectures on the WILD dataset, the stored weights can be converted into mobile-friendly models using tools provided by deep learning frameworks. Moreover, as previously specified, in our experiments, we performed only model conversion, while avoiding weight quantization so as not to alter the accuracy of the original network.

Table 3 collects stats about the considered networks. Specifically, we report the number of multiply-accumulate operations (MAC) and the frame rate (FPS) measured when deploying the converted models on an Apple iPhone XS. Measurements were gathered from processing 640×384 images and averaging over 50 consecutive inferences. To the best of our ability, all the models could run on the iPhone NPU except for MiDaS, which was not able in our tests to leverage such accelerator. On top of that, we report the performance achieved by MiDaS, showing that it requires about 5 s on a smartphone to process a single depth map, performing about 170 billion operations. This evidence highlights how, despite being far more accurate, as shown before, this vast network is not suited at all for real-time processing on mobile devices. Moving on to more compact models, we can notice how MonoDepth2 reaches nearly 10 FPS, performing one order of magnitude fewer operations. DSNet and PyDNet both perform about 9 billion operations, but the latter allows for much faster inference, at close to 60 FPS, and is about 6 times faster than previous models. Since the number of operations was almost the same for DSNet and PyDNet, we ascribe this performance discrepancy to the low-level optimization of some specific modules. Finally, FastDepth performed three times fewer operations, yet ran slightly slower than PyDNet when deployed with the same degree of optimization as the other networks on the iPhone XS.

Summarizing the performance analysis reported in this section and the previous accuracy assessment concerning the deployment of single image depth estimation in the wild, our experiments highlight PyDNet as the best trade-off between accuracy and speed when targeting handheld devices.

A video showing the deployment of PyDNet with an iPhone XS framing an urban environment is available at https://www.youtube.com/watch?v=LRfGablYZNw. At the following link is also available a PyDNet web demo with client-side inference carried out by TensorFlow JS: filippoaleotti.github.io/demo_live.

## 9. Applications of Single Image Depth Estimation

We assessed the performances of the networks. We present two well-known applications that can significantly take advantage of dense single image depth estimation. For these experiments, we used the PyDNet model trained on the WILD dataset, as described in previous sections.

**Figure 6:**
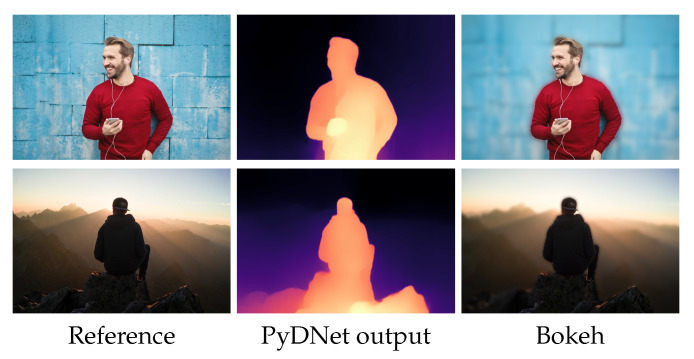
Bokeh effect. Given the reference image, we smooth farther pixels in the image using depth values provided by PyDNet.

Bokeh effect. The first application consists of a bokeh filter, aimed at blurring an image according to the distance from the camera. More precisely, in our implementation, given a threshold τ, all the pixels with a relative inverse depth smaller than τ are blurred by a 25 × 25 Gaussian kernel. In Figure 6, we show the bokeh effect appliedto single images sampled from the web, for which neither stereo pairs nor video sequences are available.

**Figure 7:**
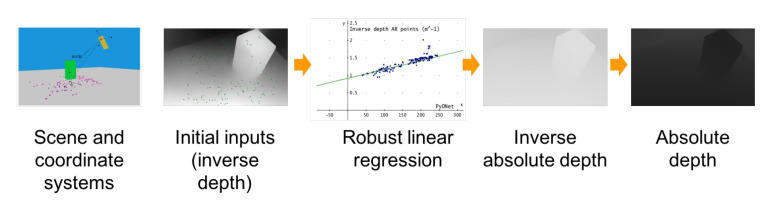
Augmented Reality (AR) pipeline. The leftmost figure depicts an object (green box) framed by a device (yellow box) capable of providing sparse absolute depth points (in purple) through the AR framework and a dense inverse depth map thanks to the monocular network. To realize our improved AR application, we move first sparse depth measurements into the inverse depth domain (second image from the left). Then, we obtain the shift and scale factor that maps such sparse points and corresponding predictions picked by the dense inverse map provided to the network by robustly regressing a linear model within a RANSAC framework, as illustrated in the chart. Finally, we scale the dense inverse map accordingly before turning back into the depth domain (respectively, the two rightmost maps) to render virtual objects consistent with the inferred geometry of the scene.

Augmented reality with depth-aware occlusion handling. Modern augmented reality (AR) frameworks for smartphones allow robust and consistent integration of virtual objects on flat areas using camera tracking with respect to fixed a reference system located in an anchor point. This goal is achieved by matching sparse feature points and taking advantage of the sensor suite (comprising accelerometers, gyroscope, etcetera) available on mobile devices. An additional outcome of this process is a number of sparse depth measurements appropriately scaled. The leftmost image of Figure 7 illustrates the standard output and reference system of a standard AR framework.

However, these frameworks miserably fail when the scene contains occluding objects protruding from the flat surfaces. Therefore, in AR scenarios, dense depth estimation is paramount to handle properly physic interactions with the real world, such as occlusions. Although some authors proposed densifying the sparse depth measurements provided by AR frameworks, it is worth observing that dynamic objects or other factors in the sensed scene may lead to incorrect depth estimations [55].

On the other hand, we argue that single image depth estimation may enable full perception of the scene suited for many real-world use cases, while potentially avoiding at all the issues outlined so far. The only remaining issue, concerned with the unknown scale factor intrinsic in a monocular system, can be robustly addressed leveraging the sparse absolute depth measurements provided by standard AR framework. Purposely, we developed a mobile application capable of handling in real-time object occlusions by combining sparse clues provided by standard AR frameworks, such as ARCore or ARKit, to scale accordingly at each frame the dense depth prediction provided by a lightweight monocular network. To achieve this goal, at first, we convert the sparse absolute depth measurements inferred by the AR framework into inverse depths to be compliant with the output domain of the monocular network, encoding an inverse depth up to a scale factor. Then, within a RANSAC framework, we regress the parameters of a linear model enabling to scale the whole network’s output according to the sparse yet scaled inverse depth predictions. Finally, we turn inverse depths into depths, obtaining absolute depth predictions enabling rendering of virtual objects consistent with the geometry of the scene. The overall pipeline outlined is illustrated in Figure 7.

**Figure 8:**
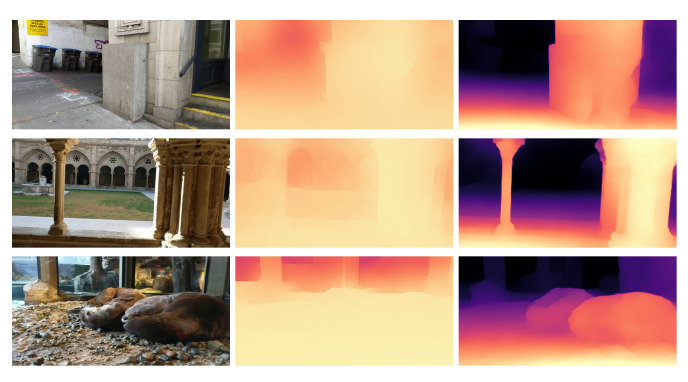
Qualitative comparison with other occlusion-aware AR methods. From left to right, the input image; the depths from [55] and PyDNet predictions.

**Figure 9:**
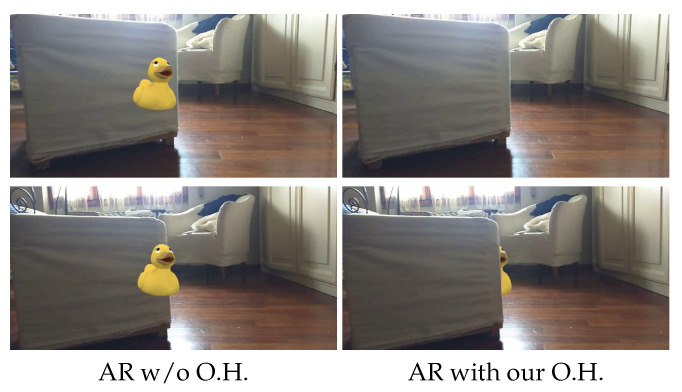
AR with occlusion handling. On the left, vanilla AR enabled by an Android device with ARCore. On the right, instead, our depth-aware AR enabled by single image depth prediction with PyDNet for occlusion handling.

Differently from other approaches, such as [55,56], our networks do not require SLAM points to infer dense depth maps, nor a fine-tuning of the network on the input video data. In our case, a single image and at least two points in scale suffice to obtain absolute dense depth perception. Consequently, we do not rely on other techniques (e.g., optical flow or edge localization) in our whole pipeline for AR. Nevertheless, it can be noticed in Figure 8 how our strategy coupled with PyDNet can produce competitive and detailed depth maps leveraging a single RGB image only. Figure 9 shows some qualitative examples of an AR application, i.e., visualization of a virtual duck in the observed scene. Once positioned on a surface, we can notice how foreground elements do not correctly hide it without proper occlusion handling. In contrast, our strategy allows for a more realistic experience, thanks to the dense and robust depth map inferred by PyDNet and sparse anchors provided by a conventional AR framework. A video concerning two different sequences is available at this link: https://www.youtube.com/watch?v=DQPmNpMcF9Q.

## 10. Conclusions

This paper proposed a strategy to achieve robust monocular depth estimation on lightweight handheld devices characterized by severe constraints concerning power consumption and computational resources. To achieve this goal, at first, we proposed a strategy to distill from a set of single still images the knowledge from a pre-trained state-of-the-art robust network unsuited for real-time performance yet capable of generalizing very well to new environments. Following this strategy, we trained and evaluated lightweight, state-of-the-art monocular depth estimation networks capable of achieving real-time performance even on mobile devices. Our exhaustive evaluation highlights that robust and real-time depth estimation in the wild from a single image is feasible on consumer devices by adopting the framework outlined in this paper. As further proof of this achievement, we have shown its deployment in two relevant well-known consumer applications. As a future research direction, we plan to exploit temporal consistency enforceable when processing in video sequences to tackle more robustly ambiguous scenes, such as those depicted in the failure cases depicted in the paper, and improve accuracy.

## Figures and Tables

**Table 1:** Quantitative results on Eigen split. † indicates models trained according to [26] training framework, otherwise we report results provided in each original paper.

			Lower is Better		Higher is Better		
Network	Abs Rel	Sq Rel	RMSE	log RMSE	δ<1.25	$\delta <1.25^{2}$	$\delta <1.25^{3}$
PyDNet	0.153	1.363	6.030	0.252	0.789	0.918	0.963
FastDepth	-	-	-	-	-	-	-
DSNet	0.130	0.909	5.022	0.207	0.842	0.948	0.979
MonoDepth2 †	**0.132**	**1.044**	**5.142**	**0.210**	**0.845**	**0.948**	**0.977**
PyDNet†	0.154	1.307	5.556	0.229	0.812	0.932	0.970
FastDepth†	0.156	1.260	5.628	0.231	0.801	0.930	0.971
DSNet†	0.159	1.272	5.593	0.233	0.800	0.932	0.971

**Table 2:** Evaluation of generalization capability. The three groups from top to bottom report experimental results concerning, respectively, (top) TUM dataset, (middle) KITTI Eigen and (bottom) NYUv2.

				Lower Is Better		Higher Is Better		
Network	Dataset	Abs Rel	Sq Rel	RMSE	Log RMSE	δ<1.25	$\delta <1.25^{2}$	$\delta <1.25^{3}$
Ranftl et al. [17]	TUM	**0.125**	**0.148**	**0.832**	**0.195**	**0.857**	**0.944**	**0.978**
Li et al. [35]	TUM	0.135	0.158	0.852	0.209	0.826	0.942	0.975
MonoDepth2	TUM	**0.147**	**0.180**	**0.916**	**0.222**	**0.811**	**0.927**	**0.967**
PyDNet	TUM	0.166	0.210	0.978	0.244	0.767	0.921	0.955
DSNet	TUM	0.168	0.215	0.994	0.248	0.762	0.917	0.951
FastDepth	TUM	0.160	0.209	0.982	0.241	0.780	0.918	0.955
Ranftl et al. [17]	KITTI	**0.157**	**1.144**	**5.672**	**0.225**	**0.780**	**0.942**	**0.980**
Li et al. [35]	KITTI	0.227	2.081	7.841	0.325	0.621	0.854	0.939
MonoDepth2	KITTI	0.164	**1.194**	6.000	**0.239**	0.752	0.928	0.974
PyDNet	KITTI	**0.162**	1.272	6.138	**0.239**	**0.760**	0.927	0.974
DSNet	KITTI	0.164	1.203	**5.977**	**0.239**	0.754	**0.929**	**0.975**
FastDepth	KITTI	0.168	1.227	6.017	0.241	0.741	0.927	**0.975**
Ranftl et al. [17]	NYU	**0.100**	**0.061**	**0.407**	**0.132**	**0.905**	**0.984**	**0.997**
Li et al. [35]	NYU	0.149	0.116	0.560	0.189	0.782	0.958	0.992
MonoDepth2	NYU	**0.123**	**0.082**	**0.473**	**0.160**	**0.848**	**0.974**	**0.995**
PyDNet	NYU	0.130	0.091	0.493	0.168	0.827	0.969	0.994
DSNet	NYU	0.134	0.096	0.505	0.171	0.820	0.968	0.993
FastDepth	NYU	0.129	0.090	0.492	0.167	0.833	0.971	0.994

**Table 3:** Performance on smartphones. We measure both the number of multiply–accumulate operations (MAC) and the FPS of monocular networks on an iPhone XS, using an input size of 640×384, averaged on 50 inferences.

Network	MAC (G)	FPS
MiDaS	172.4	0.20
MonoDepth2	16.01	9.94
DSNet	9.48	11.05
PyDNet	9.25	58.86
FastDepth	3.61	50.31

## Data Availability

Data available in a publicly accessible repository.
